# 3D-FISH analysis of embryonic nuclei in mouse highlights several abrupt changes of nuclear organization during preimplantation development

**DOI:** 10.1186/1471-213X-12-30

**Published:** 2012-10-24

**Authors:** Tiphaine Aguirre-Lavin, Pierre Adenot, Amélie Bonnet-Garnier, Gaétan Lehmann, Renaud Fleurot, Claire Boulesteix, Pascale Debey, Nathalie Beaujean

**Affiliations:** 1 INRA, UMR1198 Biologie du Développement et Reproduction, F-78350, Jouy-en-Josas, France; 2 ENVA, F-94700, Maisons Alfort, France

**Keywords:** FISH, Heterochromatin, Centromeres, Telomeres, rDNA, Nucleolus, Nuclear organization, Embryo, Computational analysis

## Abstract

**Background:**

Embryonic development proceeds through finely tuned reprogramming of the parental genomes to form a totipotent embryo. Cells within this embryo will then differentiate and give rise to all the tissues of a new individual. Early embryonic development thus offers a particularly interesting system in which to analyze functional nuclear organization. When the organization of higher-order chromatin structures, such as pericentromeric heterochromatin, was first analyzed in mouse embryos, specific nuclear rearrangements were observed that correlated with embryonic genome activation at the 2-cell stage. However, most existing analyses have been conducted by visual observation of fluorescent images, in two dimensions or on z-stack sections/projections, but only rarely in three dimensions (3D).

**Results:**

In the present study, we used DNA fluorescent *in situ* hybridization (FISH) to localize centromeric (minor satellites), pericentromeric (major satellites), and telomeric genomic sequences throughout the preimplantation period in naturally fertilized mouse embryos (from the 1-cell to blastocyst stage). Their distribution was then analyzed in 3D on confocal image stacks, focusing on the nucleolar precursor bodies and nucleoli known to evolve rapidly throughout the first developmental stages. We used computational imaging to quantify various nuclear parameters in the 3D-FISH images, to analyze the organization of compartments of interest, and to measure physical distances between these compartments.

**Conclusions:**

The results highlight differences in nuclear organization between the two parental inherited genomes at the 1-cell stage, i.e. just after fertilization. We also found that the reprogramming of the embryonic genome, which starts at the 2-cell stage, undergoes other remarkable changes during preimplantation development, particularly at the 4-cell stage.

## Background

Specific gene expression during cell differentiation results from the concerted effects of intermingled factors: epigenetic modifications of DNA and histones, fixation of transcriptional factors, nuclear localization of genes, and the formation of higher-order chromatin structures. Indeed, over the past decade, the dynamic, temporal, and spatial organization of the eukaryotic cell nucleus has emerged as a central determinant of genome function [1-4].

When analyzing the correlation between nuclear organization and differentiation, early embryonic development offers a particularly interesting, although extremely complex, system. Upon fertilization, the highly specialized male and female gametes must be reprogrammed to form a totipotent embryo that will then differentiate and give rise to all the tissues of a new individual [5,6]. In mammals, these events occur throughout the preimplantation period (in the female reproductive tract) and are thus accessible to detailed experimental investigations, especially in the mouse model. From large-scale transcriptomic analyses performed worldwide, it is now clear that this “reprogramming” process is dependent on finely tuned mechanisms of gene regulation [7]. However, few researchers have analyzed structural and functional genome organization during early embryonic development [8-11].

Many studies focusing on epigenetic modifications have shown that, immediately after fertilization, both parental genomes undergo extensive remodeling during early cell cycles that is correlated with major modifications of gene expression [7]. However, while parental genomes are first transcriptionally silenced in zygotes after fertilization, the embryonic genome is progressively turned on: in the mouse, a "minor activation" occurs at the end of the 1-cell stage [12], followed by a "major activation" at the 2-cell stage [13]. This onset of embryonic gene expression (i.e. EGA, embryonic genome activation) is characterized by a rapid increase in the synthesis of transcripts [14]. At the same time, transcription of ribosomal DNA (rDNA) is switched off in early mouse embryos and nucleoli are not present; instead, so-called nucleolar-precursor bodies (NPBs) are formed. The reinitiation of rDNA transcription occurs at the end of the 2-cell stage, at the surface of the NPBs [15]. The first differentiation events take place later on with the physical and functional separation of the inner cell mass from the trophectoderm, at the blastocyst stage (day 3.5).

Parallel, large-scale mapping studies in somatic cells have shown that chromatin is not randomly distributed within nuclei but forms higher-order chromatin structures, some of which correlate with cell differentiation and gene activity [16-18]. For example, proximity to pericentromeric heterochromatin is generally associated with gene silencing [19,20]. Centromeric and pericentromeric heterochromatic regions are highly important for chromosome stability and proper segregation [21]. However, during interphase, these regions form higher-order chromatin structures – the so-called “chromocenter” clusters [22] – that act as transcriptionally repressive structures for genes spatially located in their vicinity [16,23]. Similarly, it has been found that silencing of rDNA genes is tightly linked to heterochromatin formation [24].

When higher-order chromatin structures such as pericentromeric heterochromatin were first analyzed in the mouse, a specific nuclear architecture exclusive to the first embryonic cleavages was observed [8,9]. Decondensation of pericentromeric heterochromatin seems to take place rapidly after fertilization, and it has been suggested that this maintains transcriptional silencing until EGA [10]. Thereafter, reorganization of the centromeric and pericentromeric heterochromatin into “chromocenters” occurs concomitantly with the major phase of EGA [8-10]. In fact, interference with the reprogramming of the pericentromeric structures significantly alters development; it has been shown that disruption of chromocenters in mouse fertilized embryos results in developmental arrest [11,25] and that cloned embryos produced by nuclear transfer often show aberrant nuclear architectures with remnants of somatic-like chromocenters, correlating with poor developmental rates [9,26,27].

Most of these results were acquired through the use of immuno-fluorescence and fluorescence *in situ* hybridization (FISH) to label compartments of interest in embryos. However, one important limitation of these studies is that the analysis of the corresponding fluorescent images is mostly visual and focused on large-scale nuclear movements, which are easier to evaluate. Genome wide approaches, especially chromosome conformation capture (3C), can provide more details to help decipher key nuclear events at the molecular level [4], but their use in embryos is limited due to the small size/number of the samples.

Fluorescent imaging offers us the advantage of following several structures within each embryo, thanks to high-resolution microscopy and the combination of several color channels. However, most analyses are done either in two dimensions or on z-stack sections/projections, and only rarely in three dimensions (3D) because they would be much more time-consuming. A promising approach to explore the embryonic nucleus in more detail is the use of computational imaging [28]. At present, we are still at the very beginning of this approach, and the tools required to locate compartments of interest, to analyze their movements, and to measure physical distances still need improvement. Using this technique, however, Koehler and collaborators were recently able to describe, for the first time, 3D rearrangements of chromosome territories in preimplantation embryos [29]. We similarly analyzed major 3D nuclear rearrangements of centromeric and pericentromeric heterochromatin in bovine and rabbit embryos with dedicated computational programs [30,31].

To obtain a more complete understanding of the nuclear reorganization that takes place during the early developmental stages in mouse, we analyzed, in detail, centromeric and pericentromeric chromatin local reprogramming in preimplantation embryos with preserved 3D-shapes (from the 1-cell to blastocyst stage). We also developed new image analysis tools to quantify various nuclear parameters of the 3D-FISH images, i.e., the nuclear volume, the number of NPBs/nucleoli, the nuclear polarity, the number and shape of pericentromeric heterochromatin structures, and their proximity to NPBs/nucleoli.

Our results highlight differences in nuclear organization in paternal and maternal inherited genomes at the 1-cell stage. We also find that the reprogramming of the embryonic genome, which starts at the 2-cell stage, undergoes several abrupt changes during preimplantation development.

## Results

### Unique nuclear organization of zygotes

We first analyzed the distribution of centromeric (minor satellite) and pericentromeric (major satellite) heterochromatin in zygotes throughout the first cell cycle after fertilization (1-cell stage). At that stage, the parental genomes are separated in two haploid pronuclei (PN) containing nonfunctional NPBs, and zygotes can be classified in substages from PN0 to PN5 [32,33]. As previously described in the literature, we observed markedly different reorganizations within the male and female pronuclei from PN0 to PN5. Just after fertilization, pericentromeres organized rapidly around the NPBs in the female pronucleus (fPN; maternally inherited genome) whereas in the male pronucleus (mPN; paternally inherited genome), they remained associated together in more or less unorganized masses located in the center (Additional file 1: Figure S1). Remarkably, at PN3, only ~3% of the NPBs were not associated with pericentromeric signals in the fPN as opposed to almost 30% in the mPN (Table 1). We also noticed that the number of NPBs, while decreasing with time in both PNs, remained approximately twice as high in the mPN as compared to the fPN (Table 1).

**Table 1:** Distribution of pericentromeric signals in fPN and mPN during the 1-cell stage

	**Female PN (fPN)**		**Male PN (mPN)**	
	**No. of NPBs**	**No. of NPBs not associated with pericentromeric signals**	**No. of NPBs**	**No. of NPBs not associated with pericentromeric signals**
PN1/PN2	5.9 ± 2.6 ^a^	1.0 ± 1.7	13.94 ± 4.6 ^a’^	8.8 ± 8
(n = 16)				
PN3	2.8 ± 2.0 ^b^	0.08 ± 0.3	7.8 ± 4. 1 ^b’c^	2.5 ± 2.0
(n = 18)				
PN4/PN5	3.9 ± 2.3 ^b^	0.2 ± 0.8	5.7 ± 3 ^b’c^	1.1 ± 1.1
(n = 42)				

Values with superscripts a and a’ are significantly different at p < 0.0001.

Values with superscripts b and b’, and c and c’ are significantly different at p = 0.002.

Values with superscripts a and b are significantly different at p < 0.001.

Values with superscripts c are significantly different at p = 0.05.

**Figure 1:**
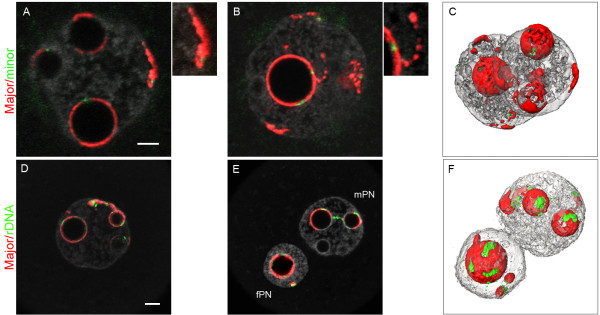
**Distribution of the pericentromeres**, **centromeres**, **and rDNA FISH signals in late 1-cell stage embryos.** 1-cell embryos at the PN4 stage (collected at 27 hphCG) were processed with pericentromeric probes (red) and either centromeric (green, upper panel A/B/C) or rDNA probes (green, lower panel D/E/F). DNA was counterstained with Yopro-1 (grey). Upper panel: **(A/B)** In both PNs, pericentromeres form more or less complete shells around the NPBs, in which the centromeres are embedded. Pericentromeres are also found at the nuclear periphery, associated with centromeric signals (see enlargement of **A**), and can form filaments with a “beads on a string” appearance (see enlargement of **B)**. **(C)** 3D reconstruction of the same nuclei. Lower panel: **(D, E)** Most of the rDNA signals are around the NPBs. However, there are occasionally some signals associated with pericentromeric filaments (extending from the NPBs towards the nuclear periphery) as well as rDNA signals joining two NPBs. **(F)** 3D reconstruction of **E**. Bar = 5 μm.

It was only in the late 1-cell stage (PN4/PN5) that pericentromeric heterochromatin adopted the same distribution in mPN and fPN, namely, more or less complete "shells" around the NPBs, in which the minor satellite centromeric signals were embedded (Figure 1A/B/C). Pericentromeric heterochromatin (filaments or more compact foci) was also found at the nuclear periphery (in 74% of fPN and 96% of mPN), in association with centromeric spots. Pericentromeric heterochromatin formed other remarkable features such as “beaded” filaments extending from the nucleolar periphery towards the nuclear periphery (Figure 1, enlargement of B). In addition, as in earlier substages (PN0-PN3), the number of NPBs remained lower in the fPNs (3.9 ± 2.3, almost all associated with pericentromeric signals) than in the mPNs (5.7 ± 3, with on average 1 of them devoid of pericentromeric signals) (n = 42, Table 1).

Owing to the tight association we observed between pericentromeric heterochromatin and the NPBs, we next analyzed the localization of rDNA sequences also known to be structurally associated with NPBs [34]. For that purpose, we performed a dual FISH with major satellite and rDNA probes (n = 66). We found most of the rDNA signals associated with pericentromeric signals at the periphery of the NPBs or within the pericentromeric filaments (Figure 1D). We sometimes noticed rDNA signals joining two NPBs (n = 4/66, Figure 1E). More surprisingly, we frequently observed rDNA foci at the nuclear periphery, associated with pericentromeric signals (Figure 1 lower panel). In fact, ~80% of the pericentromeric signals at the nuclear periphery were flanked by rDNA foci. It should also be mentioned that none of the NPBs devoid of pericentromeric signals were labeled with rDNA, and ~30% of the PNs contained NPBs bearing pericentromeric signals but no rDNA foci. It thus appears that the strong association of pericentromeric heterochromatin with NPBs is not restricted to chromosomes bearing rDNA sequences, and that such chromosomes are not exclusively associated with NPBs.

**Figure 2:**
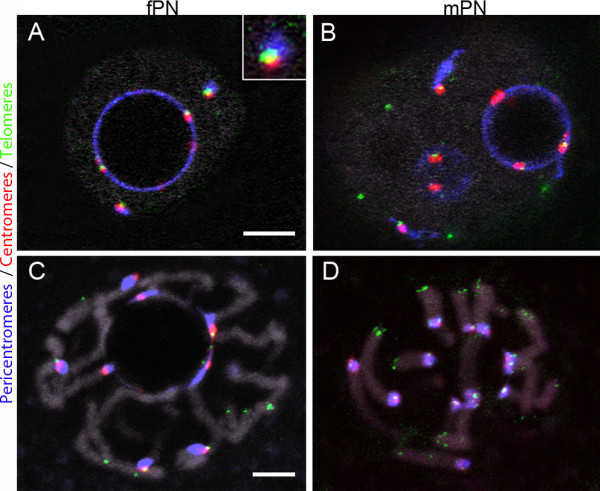
**Distribution of telomeres in late 1-cell stage embryos.** 3D-FISH was performed with telomeric (blue), pericentromeric (red), and centromeric (green) probes; DNA is in grey. **(A, B)** Single confocal images of PN5 embryos (collected at 27 hphCG). Telomeres are found 1) at the NPBs’ periphery, 2) associated with extra nucleolar centromeric/pericentromeric signals (A insert), or 3) free in the nucleoplasm **(B)**. **(C, D)** In embryos at prophase (collected at 30 hphCG), the “cartwheel” organization of condensing chromosomes can be observed in both the fPN **(C)** and mPN **(D)**. In C/D, four successive confocal sections were merged to improve chromosome visualization. Bars = 5 μm.

To gain deeper insight into chromatin higher-order organization within the pronuclei, we next analyzed the distribution of telomeres and performed triple-color FISH with major satellite, minor satellite, and telomeric probes (Figure 2). We could detect the same number of telomeric and centromeric spots in the fPN (35.0 ± 7.44 and 16.1 ± 2.0, respectively) and in the mPN (35.1 ± 6.1 and 15.7 ± 2.5, respectively) (n = 18, Table 2), which approached the expected numbers of 20 and 40, respectively. More interestingly, we found approximately half of the telomeres located around the NPBs or associated with extra nucleolar pericentromeric signals (Figure 2A) (17.7 ± 4.5 in the fPN versus 16.2 ± 5.5 in the mPN; Table 2), together with an equivalent number of centromeres. The second half appeared to be free in the nucleoplasm or close to the nuclear envelope (Figure 2B). At the end of the 1-cell stage, chromosomes condensed in both PNs through a process equivalent to prometaphase [35,36]; pericentromeres previously forming the peri-NPB shell condensed with their corresponding centromeres and anchored the chromosomes to the NPB, whereas the rest of the chromosomal scaffold extended outwards, like a “cartwheel” (Figure 2C/D) [8]. One to three chromosomes seemed to escape from this radial organization and remained at the periphery of the cartwheel. They most probably corresponded to the few centromeric/pericentromeric filaments and foci observed at the nuclear periphery from the PN3 to PN5 stages.

**Table 2:** Distribution of centromeres, pericentromeric heterochromatin, and telomeres in late 1-cell embryos (PN4/PN5 stages)

	**No. of NPBs**	**No. of centromeric spots**	**No. of centromeric spots not associated with NPBs**	**Total no. of telomeric spots**	**No. of telomeric spots associated with NPBs**	**No. of telomeric spots not associated with NPBs**
fPN	3.4 ± 2.15	16.1 ± 2.0	1.15 ± 1.14	35.0 ± 7.4	17.7 ± 4.5	15.78 ± 5.55
	(n = 30)	(n = 13)	(n = 13)	(n = 18)	(n = 18)	(n = 18)
mPN	4.9 ± 2.8	15.7 ± 2.5	1.08 ± 1.12	35.1 ± 6.1	16.2 ± 5.5	17.6 ± 5.27
	(n = 30)	(n = 13)	(n = 13)	(n = 18)	(n = 18)	(n = 18)

### Post-zygotic changes in nuclear organization

**Figure 3:**
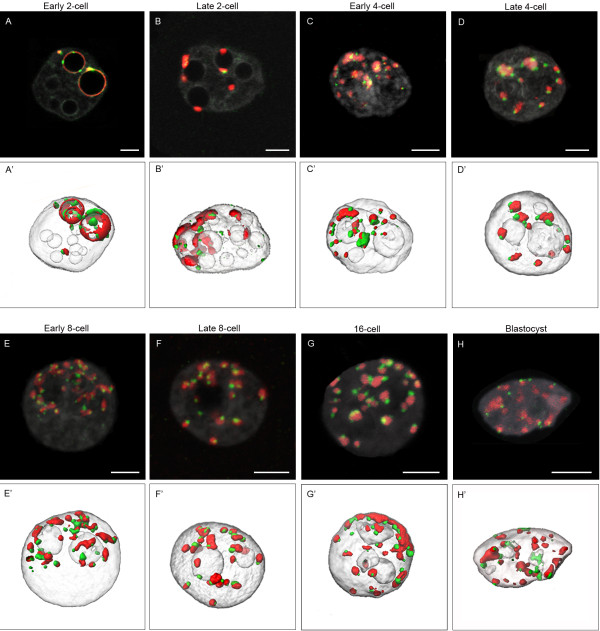
**Distribution of pericentromeres and centromeres at different stages of mouse preimplantation development**. Single confocal sections **(A-H)** of each preimplantation stage with pericentromeric (major satellite, red), centromeric (minor satellite, green), and DNA (grey) labeling are presented here, as well as the corresponding 3D reconstructions **(A'-H')**. **(A, A')** Example of an embryo at the early 2-cell stage. Pericentromeres and centromeres are essentially located at the periphery of NPBs, but some NPBs remain devoid of any signal. **(B, B')** In embryos at the late 2-cell stage, pericentromeric signals are now forming more spherical patches mostly associated to NPBs. The following images show embryos cultured up to the 4-cell (early **C**, **C’** versus late **D**, **D'**), 8-cell (early **E**, **E'** versus late **F**, **F’**), 16-cell (**G**, **G'**) and blastocyst (H, H’) stage. Pericentromeric and centromeric heterochromatin now forms chromocenters of heterogenous sizes and shapes. Note that at the blastocyst stage we randomly analyzed the trophectoderm cells and the inner cell mass. Bars = 5 μm.

After the zygotic stage, the embryonic genome undergoes structural and functional changes. For example, it is well-known that the compaction of pericentromeric heterochromatin that forms chromocenters starts at the 2-cell stage [8-10]. However, few data exist on the global nuclear morphological changes occurring during pre-implantation development, up to the blastocyst stage. We therefore performed systematic 3D-FISH with minor and major satellite probes (centromeric and pericentromeric heterochromatin). We analyzed embryos at various time-points during the 2-cell/4-cell/8-cell/16-cell/morula and blastocyst stages. Representative examples are shown in Figure 3.

We observed that remodelling of the embryonic genome indeed started at the 2-cell stage. At the beginning of the second cell cycle, the major satellites were essentially associated with the NPBs, as in zygotes, forming either thick partial rims (58% of the NPBs; n = 20) or more spherical patches (5% NPBs) (Table 3 and Figure 3A). Centromeric spots were always associated with these rims and patches (Figure 3A/A’). The remaining NPBs (37%) were free of any FISH signal (Table 3). However, by the end of the second cell cycle, the percentage of NPBs associated with spherical patches of pericentromeric heterochromatin increased (37.6%, n = 19), whereas NPBs surrounded by rims tended to disappear (24.2%) (Table 3 and Figure 3B/B’). In these embryos, most of the rDNA signals were located in close proximity to the NPBs and the pericentromeric signals, as found in zygotes (data not shown &[34]).

**Table 3:** Organization of pericentric heterochromatin in early and late 2-cell embryos

	**No. of NPBs**	**No. of NPBs not associated with pericentromeric signals**	**No. of NPBs associated with pericentromeric rims**	**No. of NPBs associated with condensed pericentromeric patches**	**No. of “free” cytoplasmic pericentromeric patches**
early 2-cell (n = 20)	13.75 ± 3.78	4.95 ± 1.76 (37%) ^b^	8.10 ± 3.26 (58%) ^a^	0.7 ± 0.57 (5%) ^a^	2.7 ± 1.7 ^b^
late 2-cell (n = 19)	11.78 ± 3.47	3.47 ± 1.54 (29.6%) ^b’^	2.67 ± 1.64 (24.16%) ^a’^	4.83 ± 2.4 (37.6%) ^a’^	4.05 ± 2 ^b’^

Values with superscripts a and a’ are significantly different at p < 0.0001.

Values with superscripts b and b’ differ at p = 0.02.

Remarkably, higher-order chromatin reorganization continued beyond the 2-cell stage. New structures containing centromeric and pericentromeric heterochromatin appeared at the 4-cell stage, forming structures very similar to classical chromocenters, i.e., a compact mass of pericentromeric heterochromatin surrounded by individual centromeres (Figure 3C/C’ and D/D’). During the same period, the number of nucleoli, which were now fully active [15], underwent an abrupt decrease between early 4-cell (11.4 ± 4.4; n = 55) and late 4-cell stages (3.0 ± 1.8; n = 94). Finally, by the blastocyst stage, the overall nuclear organization was very similar to that of somatic cell nuclei in terms of nucleoli numbers and chromocenter organization (Figure 3 from E/E’ to H/H’) [22,37].

However, we scanned more than 1000 embryos in total, making the image analysis tedious. In these conditions, only the most obvious large-scale nuclear movements could be evaluated by visual analysis. We therefore configured semi-automated image analysis tools particularly adapted to the size and geometry of the embryonic nuclei, describing quantitative morphometric features of the nuclei and the NPBs/nucleoli. We also analyzed, in detail, heterochromatin behavior in the context of such morphological changes.

### Morphometric features of nuclei and NPBs/nucleoli

**Figure 4:**
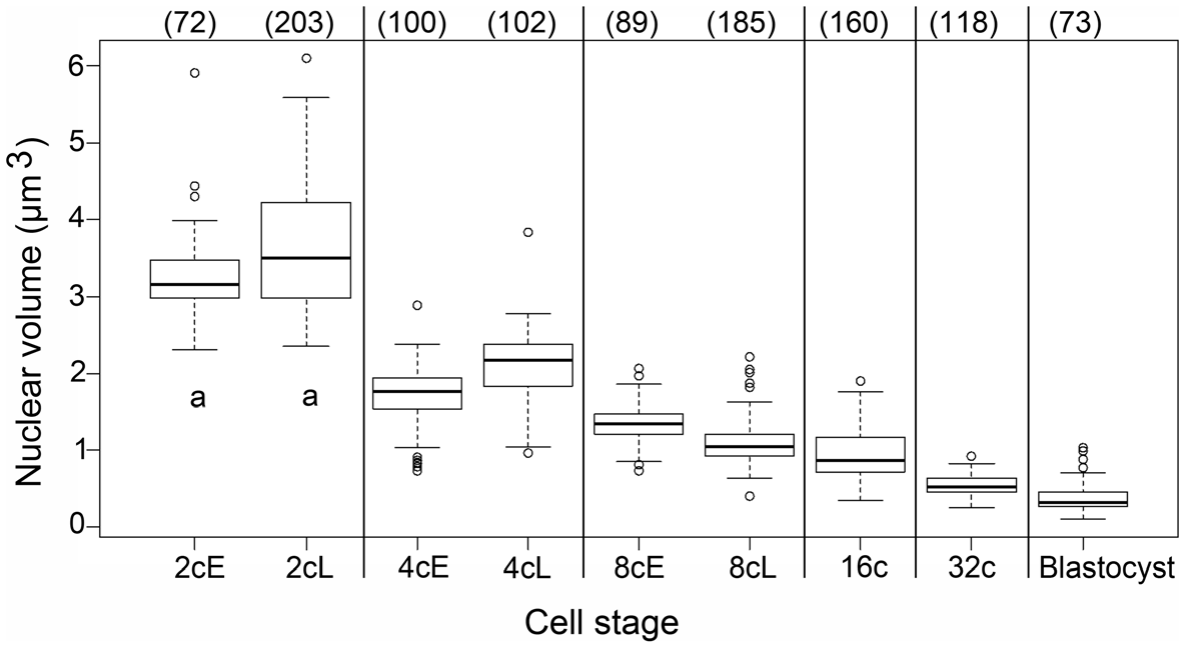
**Quantitative analysis of nuclear volume in preimplantation mouse embryos.** Box plots of the nuclear volume at each developmental stage are presented, indicating the smallest observation (sample minimum), the lower quartile, the median value, the upper quartile, and the largest observation. The number of nuclei analyzed at each stage is indicated in brackets above the box plots. At the 2-cell, 4-cell, and 8-cell stages, early (E) and late (L) embryos have been analyzed separately. Differences in mean values between each stage are highly significant, with p < 0.0001 (no subscripts) or p < 0.001 (subscript a, early and late 2-cell stage).

DNA labeling was used to delineate the embryonic nuclei from the confocal 3D-stacks and to calculate nuclear volumes (as described in Material and Methods). It should be mentioned that, for early stages, we distinguished early and late time points. However, at later stages, cellular divisions were no longer synchronous and such an analysis could not be performed; we thus pooled the data within each stage. Figure 4 shows that the nuclear volume decreased progressively from the 2-cell stage (3484 μm^3^ ± 480 μm^3^; n = 275) to the blastocyst stage (389 μm^3^ ± 193 μm^3^; n = 73) by a factor of 10, with the most marked decrease occurring between the 2- and 4-cell stages (twofold).

We next performed a quantitative automated analysis of NPB/nucleolus numbers and volumes.

**Figure 5:**
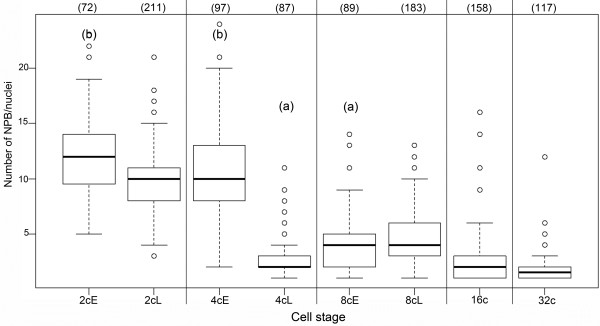
**Quantitative automated analysis of NPBs/nucleoli numbers in preimplantation mouse embryos.** Box plots of the number of NPBs/nucleoli in 2-cell through 32-cell stages (early (E) and late (L) embryos have been analyzed separately at the 2-cell, 4-cell, and 8-cell stages). The number of nuclei analyzed at each stage is indicated in brackets above the box plots. Differences in mean values between each stage are highly significant, with p < 0.0001; significant between late 4-cell and 16-cell stages (subscript a), with p = 0.00125; and less significant between early 2-cell and early 4-cell stages (subscript b), with p = 0.0103. The difference between late 2-cell and early 4-cell stages as well as between 16-cell and 32-cell stages is not significant (p > 0.05).

As shown in Figure 5 and Table 4, the number of NPBs decreased slightly but significantly between the early and late 2-cell stage (mean values 12.2 ± 3.90, n = 72 versus 9.9 ± 3.07, n = 211; p < 0.0001). This decrease during the 2-cell stage was accompanied by a marked modification (p < 0.0001) in the distribution of NPB volumes (Table 4): the median value increased from 28.7 μm^3^ (n = 887) to 41.5 μm^3^ (n = 1959). Interestingly, NPBs associated with pericentromeric heterochromatin were larger than those not associated with pericentromeric heterochromatin, both at early (p < 0.01) and late (p < 0.0001) stages. At 8 cell, the changes in nucleolar number and size distribution are much smaller, suggesting that this fusion process is less prominent.

**Table 4:** Characterization of NPBs/nucleoli in mouse embryonic nuclei

**Stage**	**No. of nuclei**	**No. of NPBs/nucleoli**	**Mean no. of NPBs/nucleoli per nuclei**	**Relative volume of NPBs/nucleoli (% of nuclear volume)**	**Median volumes of NPBs/nucleoli (μm**^**3**^**) [1**^**st**^**quartile; 3**^**rd**^**quartile]**
Early 2-cell	72	887	12.2 ± 3.90 ^a^	14.9 ± 3.93 ^ab^	28.7 ^a^
					[16.0; 50.2]
Late 2-cell	211	1959	9.9 ± 3.07 ^b^	15.6 ± 3.68 ^a^	41.5 ^b^
					[16.9; 78.9]
Early 4-cell	100	1091	10.9 ± 4.19 ^ab^	14.1 ± 4.47 ^bc^	12.1 ^cd^
					[6.2; 24.4]
Late 4-cell	87	275	2.7 ± 1.9 ^c^	14.3 ± 3.06 ^c^	64.5 ^e^
					[13.4; 203.9]
Early 8-cell	89	364	4.1 ± 2.62 ^d^	13.3 ± 2.32 ^cd^	13.4 ^cf^
					[5.5; 58.3]
Late 8-cell	184	883	4.7 ± 2.65 ^d^	12.6 ± 2.39 ^d^	11.1 ^d^
					[4.3; 33.0]
16-cell	158	283	2.2 ± 2.13 ^e^	10.8 ± 2.93 ^e^	26.1 ^af^
					[4.9; 81.0]
32-cell	117	220	1.9 ± 1.41 ^e^	9.6 ± 2.87 ^f^	19.6 ^cd^
					[3.4; 50.9]

Within each column, values with different subscripts are significantly different at p < 0.01.

In the following stage, the number of NPBs decreased drastically, as expected, from ~11 NPBs in early 4-cell (n = 100) to ~3 in late 4-cell embryos (n = 87, p < 0.0001, Table 4 and Figure 5). Remarkably, the median value of the NPB volume reached 64.5 μm^3^ by the end of the 4-cell stage (n = 275, Table 4), suggesting that the number of NPBs decreases via NPB fusion.

Controls performed on representative 2-cell and 4-cell embryos showed a slight divergence in the results obtained from manual counting of the NPBs and those from the computerized image analysis, with 8% to 10% divergence in late 2-cell (n = 101), early 2-cell (n = 39), 4-cell (n = 104) stages. However, we observed that this difference was related to the smallest NPBs only (volume < 5 μm^3^).

During later development, the number of nucleoli remained quite low (Figure 5), with a slight increase at the 8-cell stage (4.1 ± 2.61, n = 89 at early 8-cell and 4.7 ± 1.91, n = 184 at late 8-cell). However, we noticed that the total nucleolar volume relative to the nuclear volume decreased continuously from 15% at the 2-cell stage (n = 287) to approximately 10% at the 16-cell (n = 158) and 32-cell stages (n = 117) (Table 4).

### Pericentromeric heterochromatin structure and organization

**Figure 6:**
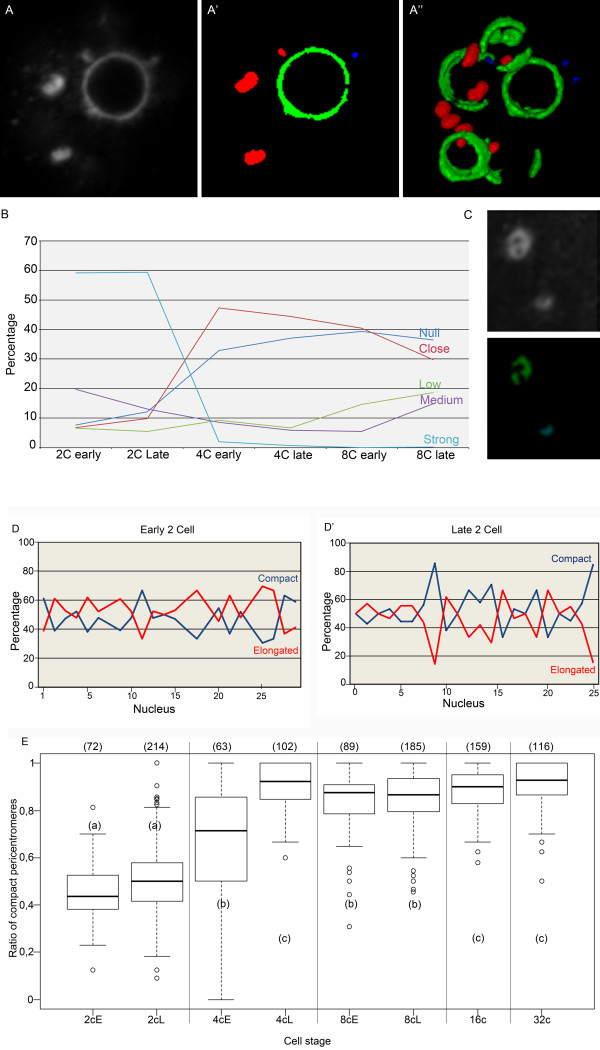
**Computerized analysis of pericentromere structures and organization in preimplantation mouse embryos.** Panel **A-A”:** Segmentation and classification of the pericentromeric signals in a late 2-cell nucleus. **(A)** Original confocal section of the 3D-FISH analysis with the pericentromeric probe; **(A’)** Same confocal section after segmentation and classification into “compact” (red), “elongated” (green), or “non analyzed” (blue) signals; **(A”)** 3D reconstruction of the pericentromeric signals after segmentation and classification. Panel **B:** Proximity between elongated pericentromeres and NPBs/nucleoli was analyzed, and five different categories were distinguished: Null, Close, Low, Medium, and Strong. The graph represents the percentage of each group at 2-cell, 4-cell, and 8-cell stages. Panel **C:** Example of a pericentromeric signal classified as “elongated” through computerized analysis, although it would be classified as “compact” by visual analysis. Note the less intense “core” of this pericentromeric signal. Panel **D-D’:** Percentage of “compact” (blue) and “elongated” pericentromeres in several nuclei from early versus late 2-cell stage embryos. Panel **E:** Box plots representing the ratio of “compact” pericentromeres relative to the total observed pericentromeric signals from 2-cell to 32-cell stages (early **(E)** and late **(L)** embryos have been analyzed separately at the 2-cell, 4-cell, and 8-cell stages). The number of nuclei analyzed at each stage is indicated in brackets above the box plots. Differences in mean values between stages with different subscripts are highly significant (p < 0.0001) to significant (p = 0.0079).

One of the major events affecting centromeres and pericentromeres during preimplantation development is their relocation from the periphery of the NPBs towards the nucleoplasm, where they form structures resembling the chromocenters in somatic mouse nuclei. In order to gain deeper insight into this phenomenon, we analyzed the “roundness” of the pericentromeric 3D-FISH signals: in brief, the surface of the object undergoing segmentation was divided by the surface of a sphere with an equivalent volume (see Material and Methods for details). We could thereafter classify the pericentromeres either as “compact”, when their roundness was greater than 0.8 and their size larger than 0.47 μm^3^, or “elongated”, when their roundness was less than 0.8 and their size larger than 0.47 μm^3^. Pericentromeric signals of less than 0.47 μm^3^ were “not analyzed” (NA); these represented less than 5% of the total volume of pericentromeres at 2-cell and 4-cell stages and less than 10% in later stages. Figure 6A/A’/A” illustrates the segmentation and classification of the signals obtained using 3D-FISH with pericentromeric probes on a 2-cell stage embryo.

We immediately noticed that pericentromeres partially surrounding NPBs usually had a roundness that was less than 0.5. We therefore created another tool to analyze the relationship between elongated pericentromeres and NPBs/nucleoli. In brief, we measured the volume of pericentromeric signal interactions with NPBs/nucleoli within a three-pixel distance from the NBPs/nucleoli (see Material and Methods for details). We then determined five categories: Null or Close for those without clear interactions versus Low, Medium, and Strong for those with pericentromere and NPB/nucleolus interactions. Figure 6B shows that the proportion of elongated pericentromeres with a strong NPB/nucleolus interaction was higher in early 2-cell than in late 2-cell embryos. It then decreased dramatically between the 2- and 4-cell stages, suggesting that the dissociation of pericentromeric heterochromatin from NBPs/nucleoli begins at the 2-cell stage and finishes at the 4-cell stage. Interestingly, in some late 2-cell nuclei, we noticed apparently “compact” pericentromeres with a less intense “core”. However, these pericentromeres were classified as “elongated” due to their crescent shape after segmentation (Figure 6C). We believe these pericentromeres represent intermediate configurations between elongated and compact heterochromatin structures.

Both elongated and compact heterochromatin structures were present in 2-cell stage embryos. Figure 6D/D’ shows the percentage of each structure in nuclei of early 2-cell and late 2-cell stage embryos. Although these percentages vary from nucleus to nucleus, we could already observe important changes during the 2-cell stage: the percentage of compact pericentromeres was below 50% in the majority of the early 2-cell nuclei and above 50% in the majority of late 2-cell nuclei. When we analyzed a larger number of nuclei from different experiments, we could still see that the percentage of “compact” pericentromeres increased between early and late 2-cell stages (p = 0.160; Figure 6E). However, it clearly appears that this number increased more sharply between the 2-cell and 4-cell stages, when it reached 90%, a value that did not vary much thereafter (Figure 6E).

### Maintenance of nuclear polarity of embryonic nuclei during preimplantation development

**Figure 7:**
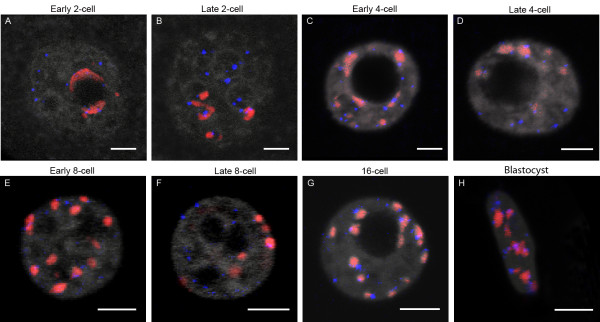
**Distribution of pericentromeres and telomeres at different stages of mouse preimplantation development.** 3D-FISH was performed with telomeric (blue) and pericentromeric (red) probes; DNA is in grey. Representative single plane confocal images are presented for: early and late 2-cell embryos **(A, B)** early and late 4-cell **(C, D)**, early and late 8-cell **(E, F)**, 16-cell **(G)**, and blastocysts **(H)**. As expected, only half of the telomeres are associated with pericentromeric signals. . Note that at the blastocyst stage we randomly analyzed the trophectoderm cells and the inner cell mass. B. Bars = 5μm.

**Figure 8:**
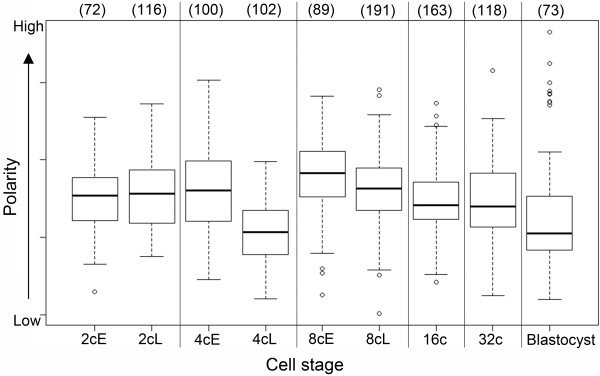
**Computerized analysis of nuclear polarity in preimplantation mouse embryos.** Box plots represent the nuclear polarity evaluation at each developmental stage from 2-cell to blastocyst (early (E) and late (L) embryos have been analyzed separately at the 2-cell, 4-cell, and 8-cell stages). The number of nuclei analyzed at each stage is indicated in brackets above the box plots.

We next performed systematic 3D-FISH with telomeric and pericentromeric probes on embryos from the 2-cell stage until blastocyst. Representative examples are shown in Figure 7. Whereas we observed a peculiar radial distribution of the telomeres versus the centromeres/pericentromeres in the 1-cell stage, their spatial distribution at the 2-cell stage was completely different (Figure 7A/B). At the later stage, centromeres/pericentromeres and their corresponding telomeres were confined to one part of the nuclei (whether associated to NPBs or as free chromocenters), while the remaining telomeres were clustered in the other part (Figure 7B). This polarity, known as Rabl-like configuration, has already been revealed in nuclei of 2-cell stage embryos via the staining of centromeres/pericentromeres [8,9,38] and in some 8-cell embryos [11]. It is believed to reflect the anaphase orientation of chromosomes and, as such, is usually lost in interphase, except in rare cases [39]. Strikingly, it seemed to be maintained in mouse preimplantation embryos during the 2-cell stage and in the following developmental stages (Figure 7D-F). As polarity is quite difficult to analyze visually *in toto* after the 4-cell stage, essentially because of the small nuclear sizes, we developed a quantitative method to evaluate polarity over a large collection of nuclei (see Material and Methods). As shown in Figure 8, centromere distribution within the nuclei highlights the presence of polarity in all stages that were examined. Curiously, we noticed that this polarity is lower in the late 4-cell stage as compared to any other stage. Polarity increases again in early 8-cell embryos, then continuously decreases up to the blastocyst stage (Figure 8).

## Discussion

Important structural remodeling and functional reprogramming affect the parental genomes during the critical preimplantation developmental period, which encompasses the transition from totipotency to differentiation. In this study, we used FISH with various genomic probes to analyze higher-order chromatin reorganization in detail on large numbers of mouse embryos with 3D preserved nuclei.

### Peculiar features of zygote nuclear organization

Just after fertilization and during the 1-cell stage, the two parental genomes are still separated in two pronuclei. This allows the observation of their different behaviors; for example, the differences in terms of epigenetic marks have already been well-documented [6,40]. Similarly, higher-order chromatin structures such as pericentromeric heterochromatin have also already been analyzed in mouse embryos [8-10]. As described in the Results section, we observed marked reorganizations within both pronuclei, male (mPN) and female (fPN), during the 1-cell stage. Just after fertilization, pericentromeres organize rapidly around the NPBs in the fPN, but remain associated in more or less unorganized masses in the mPN. Through the detailed analysis of our 3D-FISH images, we show here that paternal pericentromeric heterochromatin remains aggregated in a central mass up to the PN3 stage, and is only later dispersed to become associated with NPBs. This difference between the two parental genomes may be related to 1) the specific higher-order structure of sperm heterochromatin; 2) the progressive replacement of sperm protamines by histones; and/or 3) the specific epigenetic marks present only in male chromatin [40-43].

Regardless of their initial differences, by the end of the first cell cycle, maternal and paternal pericentromeric heterochromatin experience very similar decondensation states, together with a significant tendency to surround NPBs. This decondensation of pericentromeric heterochromatin takes place at the time of minor genome activation [12], suggesting a direct functional link between the decondensation of pericentromeric heterochromatin and the transcriptional activation of the corresponding genomic sequences.

The highly decondensed state of pericentromeres at the 1-cell stage has also been observed by electron spectroscopic imaging [44]. In our study, it is highlighted by the fact that “filaments” of pericentromeric signals could be observed escaping the periphery of the NPBs towards that of the nucleus. This result is quite surprising when compared to previous analyses performed by the immuno-staining of HP1β, the associated heterochromatin protein [8,9]. We can infer from our results that HP1β is not associated to the totality of the pericentromeric heterochromatin and is absent from the radial filaments. We believe this highly decondensed state of pericentromeres participates to the onset of pericentric satellites expression that starts in late 1-cell stage embryos [11].

It is interesting to note that a similar “dispersion” of pericentromeric heterochromatin followed by a sequential reassembly was observed upon dedifferentiation-redifferentiation of *Arabidopsis* leaf cells [45] and in nuclear transfer experiments (upon reprogramming of somatic cell nuclei by recipient oocytes) [22,27]. Taken together, these results suggest that this specific rearrangement of pericentromeric heterochromatin could be one of the features of totipotency.

### Importance of NPBs/nucleoli in global nuclear organization

In the present study, we analyzed the distribution of centromeric/pericentromeric heterochromatin with respect to the nucleolar precursor bodies and nucleoli known to evolve rapidly throughout the first developmental stages. In 1-cell and 2-cell stage embryos, we observed a tight association of this type of heterochromatin with NPBs/nucleoli, as previously described [8-10]. This tight association does not however hold for all chromosomes, since pericentromeric heterochromatin foci were also found at the nuclear periphery in interphasic 1-cell embryos (martin et al., 2006, this work) and escaping “peripheral” chromosomes are observed at condensation (Figure 2D). Whether these are specific chromosomes remains unknown; this could be analyzed by chromosome territories painting. It was, however, quite surprising to find that, whenever pericentromeres were located at the nuclear periphery, rDNA signals were almost always associated with them. This confirms that rDNA genomic sequences are not automatically associated with NPBs [34]. It also suggests that, at least in early stages, NPBs are not basic nucleolar precursors, but may have another role and/or function. This hypothesis is in agreement with the fact that oocyte nucleolar components are necessary for the reassembly of newly formed NPBs in both pronuclei after fertilization and for further embryonic development [46].

However, the exact composition of these prominent compact fibrillar structures, which are present in fully grown oocytes and early embryos, is far from being completely deciphered. Different approaches have shown that they do not contain DNA, but rather RNA, nucleolar proteins (fibrillarin, nucleolin, nucleophosmin B23), and non-nucleolar spliceosomal factors [47-51]. It is only during the first half of the 2-cell stage that components of the rDNA synthesis machinery are progressively assembled at the NPB surface, where the first rRNAs are synthesized at the mid/late-2-cell stage [15]. Remarkably, while a small but significant cell-cycle-dependent decrease in NPB number is observed at 1-cell and 2-cell stages, the decrease is more drastic in 4-cell embryos and is accompanied by a large increase in the median NPB volume. This might reflect a rapid transition in the NPBs’ function. Indeed, if the onset of rRNA synthesis was previously precisely timed [15], nothing is known concerning the dynamics of the other steps of rRNA maturation and pre-ribosomal particles assembly.

From a more structural point of view, the fact that the decrease in NPB number is associated with an increase in the median NPB volume, without a significant reduction in the overall volume, suggests the existence of a fusion process in early embryos. A similar fusion process could explain the slight decrease in NPB number at late 1-cell, as suggested by the rDNA bridges sometimes observed between 2 NPBs (Figure 1). Remarkably, the increase of the NPB volume stops at the 4-cell stage and is not observed anymore at the 8-cell stage. This would fit with the fact that active polymerase I transcription and related processing machineries are functionally organized at the NPB surface starting from the end of the 4-cell stage [15]. This fusion process of the NPB could reflect the entropy-driven nonspecific self-organizing forces (“depletion-attraction”) proposed by some authors to underlie the principles of nuclear organization [52-55]. Recent computer simulation of chromatin dynamics [56] suggests that these “depletion-attraction” forces are sufficient to explain the position of chromocenters and nucleoli in interphasic *Arabidopsis* nuclei.

### Structural features of the centromeres/pericentromeres in post-zygotic embryonic stages

Because of the highly decondensed state of pericentromeric heterochromatin in zygotes, we were not able to segment the FISH signals in these embryos with sufficient precision to perform further computational analysis. On the other hand, as reorganization of the centromeric and pericentromeric heterochromatin into “chromocenters” occurs post-zygotically, in subsequent stages we were able to more precisely analyze heterochromatin reorganization as well as various nuclear parameters using the 3D-FISH images. Unique image analysis tools developed for large objects such as individual embryos *in toto* were specifically adapted to analyze nuclear elements of highly different and complex sizes and shapes, especially the pericentromeric signals. Finally, as all the segmented signals/objects bore labels, we were able to analyze their relationships and measure interaction volumes. Thanks to these computational tools, we were able to analyze, for the first time, a large number of embryos (more than one thousand) covering the whole preimplantation period.

These methods also allowed us to statistically document development-dependent modifications of embryonic genome organization. In particular, we show here that nuclear polarity is conserved up to the 32-cell stage but decreases in blastocysts, as previously suggested by 2D-FISH on centromeric repeats [38].

Unexpectedly, we also found that the 4-cell stage represents a major step in preimplantation development. When we classified the pericentromeres as either “compact” or “elongated”, we observed that the proportion of elongated pericentromeres with a strong NPB/nucleolus interaction was higher in the early 2-cell than in the late 2-cell stage. This proportion then decreased dramatically between the 2- and 4-cell stages, while the percentage of “compact” pericentromeres increased drastically to reach 90%. Altogether, this suggests that dissociation of pericentromeric heterochromatin from NBPs/nucleoli begins at the 2-cell stage but finishes at the 4-cell stage. The factors or mechanisms that first favor pericentromeric/centromeric association to NPBs and then initiate the formation of chromocenters remain largely unknown.

However, one such factor could be the HP1β protein. In somatic cells, the presence of HP1β in fibrillarin-rich regions of nucleoli has already been reported [57]. In mouse 1- and 2-cell embryos, we previously showed that fibrillarin is located at the NPB surface [15] and could therefore represent an anchoring protein for HP1β and pericentromeric heterochromatin. Indeed, in 1-cell stage embryos, HP1β accumulations are detectable in the fPN mainly around NPBs and have been also detected, in lower amounts, in association with the paternal pericentromeres [8,10,58,59]. This hypothesis is supported by recent data showing HP1 mislocalization, abnormal nuclear organization, and developmental arrest in H3.3 K27R mutant embryos [25].

Modifications of epigenetic marks could also be involved in the reorganization of pericentromeric/centromeric chromatin. Complex and asymmetric histone/DNA modifications occur continuously throughout early development in both parental genomes and may regulate the balance between pericentromeric “elongated” versus “compact” structures [6,40].

Finally, regarding other factors that might play a role in heterochromatin assembly and organization, it must be recalled that an unidentified RNA is an integral component of pericentromeric heterochromatin in humans [60] and is necessary for the accumulation of HP1α on pericentromeric heterochromatin [61]. Moreover, small centromeric RNAs are involved in murine centromeric heterochromatin assembly [62], and in mouse embryos, small RNAs seem to participate in the formation of chromocenters as shown by the use of locked nucleic acid (LNA)-DNA gapmers to interfere with the transcription of major satellites in early embryos. This induces developmental arrest before the completion of chromocenter formation [11].

## Conclusions

Altogether, our results show that significant genome restructuring occurs during the entire preimplantation period. Just after fertilization, zygotes have a very peculiar nuclear organization with highly decondensed pericentromeric heterochromatin structures. During the next cell cycle (at the 2-cell stage), nucleolar precursor bodies (NPBs) and pericentromeric heterochromatin undergo important reorganization, as previously described in the literature. However, thanks to new computational tools, we were able to analyze these elements for the first time in a large number of embryos, all the way up to the blastocyst stage. We believe that these tools could now be used to enable detailed analyses of fluorescent 3D images in other models/organisms. Specifically, we consider computational imaging a promising approach to explore large-scale nuclear movements.

We also demonstrated that the 4-cell stage represents a major step in preimplantation development, especially with regards to pericentromeric structures. Pericentromeric structures may impact the regulation of developmental genes, particularly on heterochromatin-dependent gene silencing. As in somatic cells, the role of these rearrangements during preimplantation development may be to bring different nuclear compartments (chromocenters, nuclear periphery) in close proximity in order to activate/repress specific genes yet to be identified.

## Methods

### Ethics statement

Animal care and handling were carried out according to European regulations on animal welfare. NB has the authorization to work with laboratory animals from the departmental veterinary regulatory service (license N° 78–95) and from the local ethics committee (Comethea Jouy-en-Josas/AgroParisTech).

#### Mouse embryo collection and culture

Embryos were produced by natural fertilization of C57/CBAF1 mice. Superovulation was induced by injection of pregnant mare serum gonadotropin (PMSG, Intervet, 5 UI) followed, 48 h later, by injection of human chorionic gonadotropin (hCG, Intervet, 5 UI). Female mice were then mated with C57/CBAF1 males. Fertilization occurred at about 12 hours after hCG injection, which was used as the reference point for embryonic development (hours post-hCG i.e. hphCG). Fertilized eggs were collected at the 1-cell stage from the *ampulla* in M2 medium (Sigma) after a brief treatment with 1 mg/ml of hyaluronidase in phosphate-buffered saline (PBS, pH 7.5) to separate them from the surrounding follicular cells. *In vivo* developed 2-cell stage embryos were collected from the mice oviducts at 38 hphCG, 40 hphCG, and 48 hphCG, and immediately processed by FISH (see below). Later stages were obtained from embryos collected at the 1-cell stage and cultured *in vitro* in M16 medium (Sigma) at 37°C in a humidified atmosphere enriched to 5% CO_2._ They were processed at 53 hphCG (early 4-cell), 62 hphCG (late 4-cell), 64hphCG (early 8-cell), 72 hphCG (late 8-cell), 82 hphCG (16-cell), and 110 hphCG (blastocysts).

#### 3D-FISH

Unless otherwise specified, all steps were performed at room temperature. The zona pellucida of embryos was first removed through two rapid incubations in acidic tyrode (Sigma). The embryos were then rinsed in M2 medium, fixed in 4% paraformaldehyde (PFA) for 30 min, rinsed in PBS, and gently deposited with a minimum amount of PBS on microscope slides to allow adherence. They were then fixed again in 4% PFA for 30 min, permeabilized for 30 min in 0.5% Triton X-100, and rinsed once for 5 min in 2x saline-sodium citrate (SSC), pH 6.3. RNA digestion was performed by incubation in 200 μg/ml RNase (Sigma) in 2x SSC for 30 min at 37°C. After two rinses of 5 min each in 2x SSC at room temperature, the slide was equilibrated in the hybridization buffer (50% formamide, SSC 2X, Denhardt 1X, 40 mM NaH_2_PO_4_, 10% dextran sulfate) for 1–2 h. The probes and the slide were separately denatured for 10 min at 85°C in the hybridization buffer. We deposited the probes onto the slide, which was then placed at 37°C for 24 h in a humidified chamber. After two rinses in 2x SSC at 42°C, samples were either directly post-fixed in 2% PFA for 15 min, or further processed for immunodetection of the telomeric probes: permeabilization for 10 min in 0.5 Triton X-100, blocking for 15 min in 4x SSC containing 1% bovine serum albumin (BSA), and incubation with the secondary antibody for 45 min. DNA was counterstained with YoproI (Molecular probes, 1 μM) or propidium iodide (Sigma, 1 μg/ml).

#### FISH genomic probes

For the detection of major satellites (pericentromeric heterochromatin), we used a probe prepared by PCR on genomic mouse DNA with the primers 5’-CATATTCCAGGTCCTTCAGTGTGC-3’ and 5’-CACTTTAGGACGTGAAATATGGCG-3’, and Cy3- or Cy5-labeling by random priming (Invitrogen Kit, Ref 18095–011). Similarly, for minor satellite detection (centromeric heterochromatin), we used the following two primers: 5’-ACTCATCTAATGTTCTACAGTG-3’ and 5’-AAAACACATTCGTTGGAAACGCG-3’. For telomere detection, we used the mixmer tTaGgGtTaGgGtTaGgG [3'] Biotine, a kind gift of C. Escudé (MNHN).

The plasmids containing the cloned gene fragments of the mouse 28S rDNA (BE-2-pSP64, 1.5kb) [63] and 18S rDNA (SalC-pSP64, 2kb) [64] were provided by Pr. J. Britton-Davidian (ISEM - UMR 5554). The 28S and 18S rDNA were purified with PROMEGA Pure Yield Plasmid Miniprep System, labeled separately with Digoxigenin-11-dUTP by nick translation according to the Roche Protocol, then mixed together in the hybridization buffer.

#### Microscopy and image acquisition

All specimens were mounted in Vectashield (Vector Laboratories, Burlingame, CA, USA). To preserve the three-dimensional nuclear structure as much as possible, a thin spacer was drawn with a Dako-pen around the embryos before covering them with a 170-μm thick coverslip. Imaging was performed with an inverted Zeiss LSM 510 confocal microscope (MIMA2 platform, INRA) with a 63X oil immersion objective (Plan-Apochromat, N.A.1.4). The z-stacks were acquired using a frame size of 512 × 512, a pixel depth of 8 bits, and 0.371 μm z-steps, with sequential multitrack scanning using the 488-, 543-, and 633-nm wavelengths of the lasers.

#### Computational image analysis

All embryos were first visually analyzed with the LSM510 software, step-by-step through the confocal z-stacks, and with the help of 3D reconstructions using AMIRA software. Except for the 1-cell stage embryos, which presented a peculiar nuclear organization, we then analyzed all the preimplantation embryos with the semi-automated image processing and analysis tools described hereafter. These tools are based on the ITK library ( http://www.itk.org) interfaced with Python scripting language [65]. In each case, the LSM image files were first imported with the Bio-Formats library ( http://www.loci.wisc.edu), then the color channels were split into separate 3D data sets and upsampled to get a threefold increase in the number of pixels along the z-axis with an isotropic voxel size [0.1236 × 0.1236 × 0.1236 μm^3^. Images were then processed to get segmented, labeled objects. To check the efficiency of the segmentation procedures, segmented images were superimposed on their original grayscale image using either macros developed with the ImageJ software ( http://imagej.nih.gov/ij/) or the 3D object analysis from Fiji software ( http://fiji.sc).

#### Segmentation of nuclei

Segmentation of the nuclei in the DNA channel was a critical step because it defined the regions of interest (ROIs) where we looked for centromeric and pericentromeric structures in our 3D data sets. Since most voxels corresponded to the background of the images, a 3D binary mask was first determined by a threshold method largely used in astronomy: it analyzes only the background intensities and assigns the intensity value [mean + f * sigma] as the lower threshold [66]. To get the best mask, the weighting factor f was used as the signal-to-noise ratio (SNR) in the embryos. This SNR value was generally between 1 and 2 under our image acquisition conditions; when it was outside this range, we used the closest limit of this interval.

Next, nuclei were extracted from the binary mask with an *a priori* method based on their size and shape: a combination of 2D and 3D attribute opening transformations was applied to remove the smallest objects. Connected voxels representing nuclei were then identified with label object representation and manipulation filters [67]; 3D morphological opening and closing transformations were applied to fill and smooth the rough labeled objects. Finally, the bounding box of each nucleus was used to crop smaller 3D data sets in the three color channels, allowing both faster processing and lower computer memory requirements.

#### Segmentation of centromeric and pericentromeric signals

Segmentation of centromeric (minor satellite) and pericentromeric (major satellite) signals obtained by FISH was performed with two different, but similar, procedures. In the minor satellite 3D data sets, centromeric signals appeared quite spherical and could be extracted with a one-scale procedure defined to find spots. In the major satellite 3D data sets, pericentromeric signals appeared as different shapes and a multiscale extraction was therefore required. However, these two procedures followed common rules: 1) a preliminary step was required to prepare the cropped images for segmentation, then we had to 2) produce binary masks containing these structures, 3) label connected binary voxels in order to generate independent objects, and 4) remove some of the objects that were not biologically pertinent.

In the pre-segmentation step, the noise was eliminated from cropped images using a 2D median filter (radius = 1 pixel). The histogram of gray values was then normalized to a mean value of zero and a standard deviation equal to 1. The resulting image was rescaled between 0 and 255 before subsequent treatments. Next, we decreased the local background around the intensity peaks with a morphological top-hat transformation to produce binary masks using an intensity threshold filter set as [mean + 3.3*sigma].

Since top-hat transformation is a filtering method that generates peaks, we needed to determine which peaks really represented pericentromeric signals (“true” values). To identify the brightest regions where the structures should be present, we applied a Gaussian filter with a wide sigma value (1.24 μm) followed by an intensity threshold set as [mean + 3.3*sigma]. We then used three different structuring elements (3 × 3 × 1, 8 × 8 × 2, and 15 × 15 × 1 voxels) to find the pericentromeric signals. The binary masks created by these top-hat transformations were combined through an OR bit-wise filter to obtain one binary mask containing the intensity peaks of different sizes. The binary mask of the intensity peaks was then filtered 1) by the binary mask of the brightest regions to remove those in the darkest areas, and 2) by the ROI of the nucleus to keep only those in the nucleus. Finally, a 3D shape attribute opening transformation was applied to remove binary structures smaller than 0.123 μm^3^, i.e. a spherical volume of 5 voxels diameter. Thereafter we used the label representation filters to identify connected voxels as independent objects, and we kept only the labeled objects corresponding to “true” heterochromatin signals.

The top-hat transformation applied to the centromeric data set used a local neighborhood of 3 × 3 × 1 voxels. However, preliminary manual analysis performed with the Fiji software showed that the largest labeled objects sometimes represented the juxtaposition of two centromeric spots, and that some of the smallest labeled objects corresponded to background values. To determine parameters that could be used to improve the automatic selection, we manually analyzed several 2-cell and 4-cell nuclei with the R statistics software ( http://www.r-project.org). Consequently, three selection processes were used: first, when the labeled objects were bigger than 1.90 μm^3^, we applied morphological erosion filtering with a small isotropic structuring element (1x1x1 voxels). After the labeling of connected voxels, a dilation with the same structuring element was applied in order to recover two labeled objects when possible. Second, labeled objects with a flatness greater than 3 were removed when their volume was smaller than 1 μm^3^, and third, when the number of labeled objects was greater than 40 (i.e. the number of chromosomes in the mouse nucleus) all labeled objects smaller than 1% of the overall spots were removed.

#### Segmentation of NPBs and nucleoli

In early embryonic nuclei, NPBs and nucleoli are compact structures that appear as black round areas after DNA staining. Therefore, segmentation of NPBs/nucleoli was done by searching for dark regions within the DNA images. First, binary objects were obtained using a 2D Otsu threshold method on the cropped DNA image in which the contour of black round areas had been amplified by the addition of the gradient filtered image (sigma = 0.62 μm). Since the nuclear contour was also extracted by the Otsu method, it was necessary to discriminate binary objects connected to this nuclear contour by subtracting the nuclear contour obtained from the nuclear mask. Then we applied a morphological opening transformations that smoothed rough objects’ contours, separated collapsed objects, and removed objects smaller than 0.23 μm^3^. A modification of the segmentation workflow was necessary for 32-cell stage embryos because nucleoli were no longer spherical at that stage. The 2D Otsu method was replaced by a 3D Otsu method and the nuclear contour was then removed by searching for the minimum values between the 3D Otsu image and the nuclear binary mask.

Subsequently, we performed a preliminary manual analysis using the 3D object analysis of the Fiji software to determine criteria that could be used to remove objects showing features incompatible with nucleoli structures. These criteria were essentially based on a combination of the flatness and the elongation of the labeled objects. Different combinations were used depending whether the value for the labeled object’s volume was lower or higher than 94.5 μm^3^. In the first case, objects showing roundness (surface of the object divided by the surface of a sphere with an equivalent volume) lower than 0.5 were removed. In the second case, we noticed that, most of the time, the objects were formed by two very close nucleoli. Therefore, we applied a morphological erosion with a mild structuring element (3 × 3 × 3 voxels), and after labeling of connected voxels, a dilation with the same structuring element usually enabled us to recover two separate labeled objects.

However, when we checked our procedure using ImageJ macros to superimpose the contour of objects onto grayscale images, we noticed that NPBs/nucleoli were sometimes missing after segmentation, especially when they were too small and located on the nuclear periphery, while others were not correctly segmented. We therefore added one step to check the segmentation results manually and remove improper NPBs/nucleoli. To add missing NPBs/nucleoli, we drew a new image containing circles corresponding to the midsection of the missing NPBs/nucleoli on the superimposed image. This provided centroid positions and ellipsoidal dimensions of the NPBs/nucleoli on which to perform asymmetrical reconstruction by morphological dilation and to insert newly labeled objects using the ITK software library. Finally, when nucleolar reconstruction was not efficient enough, we discarded the corresponding nucleus from our analysis.

#### Interactions between labeled objects

The fact that each object was labeled enabled us to study the interaction between different objects, labeled “A” and “B”. The image of labeled object “A” was thresholded to obtain a binary mask that was slightly dilated with a small structuring element. This mask was then applied to the image of labeled object “B”. The resulting image contained several objects showing only the labels of object “B” intersecting with object “A”. The slight dilation of the binary mask was performed to identify “B” connected objects separated by less than 0.5 μm from an object “A”.

For example, to know which pericentromeres were connected with NPBs/nucleoli and to analyze their degree of proximity, we applied a 3D morphological dilation to the binary mask of NPBs/nucleoli with a small structuring element (3 × 3 × 3 voxels). Then we determined the labels of intersected objects with this new mask and we measured the interaction surface of pericentromeres with nucleoli on the new binary mask. To analyze the degree of interaction between elongated pericentromeres and NPBs/nucleoli, we compared the observed interaction surface with the theoretical interaction surface of a sphere with a volume equivalent to the pericentromeres’ volume. Ratios smaller than 1 corresponded to elongated pericentromeres located close to NPBs/nucleoli or showing only a weak interaction. Ratios higher than 1 indicated that these elongated pericentromeres interacted more strongly with nucleoli.

Statistical analysis and boxplot representations were performed with the R statistical software. The data from different cell stages were compared using a Wilcoxon test.

#### Nuclear polarity

The segmented images of the nuclei and the centromeres were first spatially normalized to make their principal moments equivalent. Polarity of centromere distribution was defined as the distance between the center of mass of the previously extracted centromeres and the center of mass of the nucleus and measured using the ITK software library. This distance was then normalized using the radius of a sphere of equivalent volume, and we tested whether this distance was significantly different from the value that could be obtained for a random distribution of centomeres. Random patterns were generated for each nucleus with the same number of centromeres as detected in the nucleus. The distances for the random distribution were measured for 500 independent patterns, and we were able to calculate a p-value for each nucleus using the proportion of random patterns with a distance equal to or above that observed. Under the hypothesis that centromeres are randomly distributed, the p-value within a population is uniformly distributed between 0 and 1. This was tested using a two-sided Kolmogorov-Smirnov test (α = 1%) in the R statistical software package.

## Competing interests

The authors have nothing to disclose; no competing financial interests exist.

## Authors' contributions

TAG and ABG conducted most of the experimental part of the work, especially the FISH experiments. RF and CB participated in embryo collection and FISH experiments. TAG, PA, ABG, and NB performed the confocal observations. PA and GL performed most of the data analysis. NB and PD coordinated the work. TAG, ABG, PA, PD, and NB co-wrote the manuscript. All authors read and approved the final manuscript.

## Supplementary Material

Additional file 1 **Figure S1.** Is a figure showing 3D-FISH images obtained on early 1-cell stage embryos with pericentromeric and centromeric probes. Click here for file
