# Supplementary figures and images for: 3D-FISH analysis of embryonic nuclei in mouse highlights several abrupt changes of nuclear organization during preimplantation development
Source: BMC Dev Biol. 2012 Oct 24;12:30. doi: 10.1186/1471-213X-12-30 (PMC3517311; doi:10.1186/1471-213X-12-30)

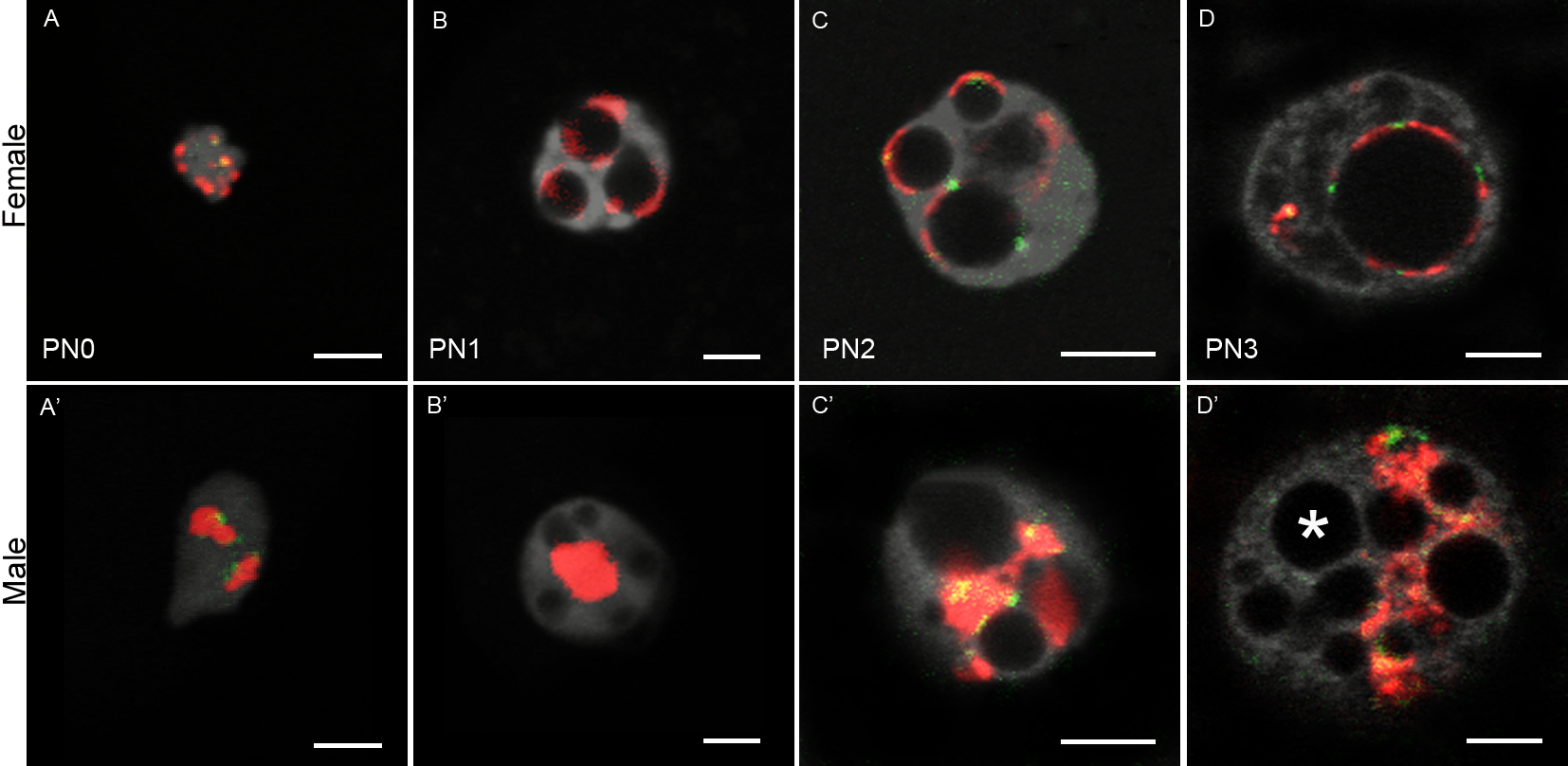

Supplement: Additional file 1 — Figure S1. Is a figure showing 3D-FISH images obtained on early 1-cell stage embryos with pericentromeric and centromeric probes. [file 1471-213X-12-30-S1.tiff]
